# Habitat of *In Vivo* Transformation Influences the Levels of Free Radical Scavengers in *Clinostomum complanatum*: Implications for Free Radical Scavenger Based Vaccines against Trematode Infections

**DOI:** 10.1371/journal.pone.0095858

**Published:** 2014-04-23

**Authors:** Atif Zafar, Asim Rizvi, Irshad Ahmad, Masood Ahmad

**Affiliations:** Department of Biochemistry, Faculty of Life Sciences, Aligarh Muslim University, Aligarh, Uttar Pradesh, India; Instituto Butantan, Brazil

## Abstract

**Background:**

Since free radical scavengers of parasite origin like glutathione-S-transferase and superoxide dismutase are being explored as prospective vaccine targets, availability of these molecules within the parasite infecting different hosts as well as different sites of infection is of considerable importance. Using *Clinostomum complanatum*, as a model helminth parasite, we analysed the effects of habitat of *in vivo* transformation on free radical scavengers of this trematode parasite.

**Methods:**

Using three different animal models for *in vivo* transformation and markedly different sites of infection, progenetic metacercaria of *C. complanatum* were transformed to adult ovigerous worms. Whole worm homogenates were used to estimate the levels of lipid peroxidation, a marker of oxidative stress and free radical scavengers.

**Results:**

Site of *in vivo* transformation was found to drastically affect the levels of free radical scavengers in this model trematode parasite. It was observed that oxygen availability at the site of infection probably influences levels of free radical scavengers in trematode parasites.

**Conclusion:**

This is the first report showing that habitat of *in vivo* transformation affects levels of free radical scavengers in trematode parasites. Since free radical scavengers are prospective vaccine targets and parasite infection at ectopic sites is common, we propose that infections at different sites, may respond differently to free radical scavenger based vaccines.

## Introduction

Trematode parasites infect all vertebrates, including humans, and cause severe morbidity and disease. *Clinostomum complanatum* (Rudolphi, 1814) has been widely used as a model species of trematodes for studies on host-parasite interactions [1]–[4] with a wide geographical distribution in tropical and sub tropical regions [5]. *C. complanatum* causes yellow grub disease in several species of fish, such as *Trichogaster fasciatus* and *Channa punctatus*, particularly in the Indian subcontinent [2] and Africa [6], leading to economic loses. *C. complanatum* has also been reported to infect oral cavity and cause laryngopharyngitis in humans, thus showing its zoonotic potential [7]–[9].

The natural definite host of *C. complanatum* are ardeiid birds, such as egrets, which feed on infected fish [10]. The progenetic metacercariae of *C. complanatum* inhabit the peritoneum of the forage fish *T. fasciatus* [11] which is essentially a hypoxic environment [2]. The adult (ovigerous) worms occur in the oxygenic environment of the buccopharayngeal region of the avian definitive host. The progenetic metacercariae attach itself to the buccal epithelium and remain tenaciously attached and voraciously feed on mucous and host blood [1].

Trematode parasites have developed several adaptive survival strategies to evade the host environment of maturation, thereby surviving for several years in the host perpetuating their life cycle. In the previous studies it has been shown that *C. complanatum* can modulate its phenotype at the ultra structural and molecular level according to its environment of maturation [12]. However, the effect of levels of oxygen on the free radical scavengers of *C. complanatum* in the microenvironment of maturation has not been reported. Characterisation of such an effect on the free radical scavengers may prove to be useful to evaluate the use of these scavengers as the promising vaccine targets against the trematode infections.

The present study was thus, carried out to see if the habitat of maturation of the progenetic metacercaria (larval stage) to adult (ovigerous) worm may have any effect on the levels of free radical scavengers of parasite origin during its *in vivo* transformation.

## Materials and Methods

All chemicals used were procured from Sigma (Saint Louis, USA). Experimental animals used were purchased locally from laboratory animal suppliers. Live field infected *T. fasciatus* was procured from the local fish market and were maintained in standard aquaria on commercially available fish food until used.

### Ethical statement for animal experimentation

Animal experimentations were permitted by Ministry of Environment and Forests, Government of India under registration no 714/02/a/CPCSEA issued by Committee for the Purpose of Control and Supervision of Experiments on Animals (CPCSEA) dated 16th November, 2002 and approved by the institutional ethical committee of Department of Biochemistry, Aligarh Muslim University, Aligarh, India.

### Isolation of progenetic metacercaria of *C. complanatum*

*C. complanatum* progenetic metacercariae were isolated aseptically from the peritoneum of the forage fish *T. fasciatus* and transferred into 50 mM phosphate buffered saline (PBS) pH 7.0, and rinsed in PBS containing 1% streptomycin and penicillin G, and subsequently washed in several changes of PBS quickly.

### Establishment of infection in chickens and parasite recovery

Infection was established in one day old leghorn chickens by aseptically transferring the parasites to the buccal cavity using a soft paint brush, using the method of Abidi and Nizami [1]. The chickens were fed on grounded corn and water provided *ad libatum*. The worms were harvested on day one to four, post infection, using a fine forceps and a soft paint brush from the oral cavity after sacrificing the birds by cervical dislocation. The worms were transferred into 50 mM PBS pH 7.0, and rinsed in PBS containing 1% streptomycin and penicillin G, and subsequently washed in several changes of PBS quickly.

### Establishment of infection in rabbits and parasite recovery

Adult male New Zealand white rabbits were used for the experiments. Infection was established according to the protocol of Rizvi et al. [3]. The attached adult worms were harvested from the site of infection after sacrificing the animal by cervical dislocation. The worms were transferred into 50 mM PBS pH 7.0, and rinsed in PBS containing 1% streptomycin and penicillin G, and subsequently washed in several changes of PBS quickly.

### Establishment of infection in rats and parasite recovery

Adult wistar rats of about 100±20 grams were maintained on standard rat chow and water provided *ad libatum* in polypropylene cages. Infection was established using the protocol of Rizvi [13]. Worms were harvested and transferred into 50 mM PBS pH 7.0, and rinsed in PBS containing 1% streptomycin and penicillin G, and subsequently washed in several changes of PBS quickly.

### Homogenate preparation

Homogenate was prepared essentially according to Rizvi et al. [2] with minor modifications. The fresh progenetic metacercaria and the harvested worms were homogenised in a total volume of 3 ml, 50 mM phosphate buffered saline, pH 7.0, in a pre chilled pestle and mortar. The homogenate was centrifuged at 5000 rpm for 10 min at 4°C and the supernatant was stored at −20°C till used.

### Protein estimation

The protein in the samples was determined by the modified dye binding method using bovine serum albumin as standard [14].

### Thiobarbituric acid reactive substances (Lipid peroxidation)

Thiobarbituric Acid Reactive Substances (TBARS) were estimated in the homogenate by the method of Buege and Aust [15], with minor modifications. To 1.5 ml reaction mixture, 0.5 ml of 10% TCA (trichloroacetic acid) and 0.5 ml of 0.6 M TBA (2-thiobarbituric acid) were added and the mixture was incubated in a boiling water bath for 20 minutes. The absorbance was read at 532 nm and converted to nmoles of TBA reactive substances using the molar extinction coefficient.

### Estimation of superoxide dismutase (SOD) (E.C.1.15.1.1)

SOD was assayed by the method of Marklund and Marklund [16] based on the ability of superoxide distmutase to inhibit the auto-oxidation of pyrogallol. Reaction mixture in a final volume of 3.0 ml containing 50 µl of sample with 2.85 ml of tris-succinate buffer (0.05 M, pH 8.2) was incubated at 25°C for 20 minutes. The reaction was initiated by the addition of 100 µl of 8 mM pyrogallol and the change in absorbance was measured at 412 nm for 3 minutes. The specific activity was reported in units per mg of protein. One enzyme unit was defined as the amount of enzyme required to cause 50% inhibition of auto-oxidation of pyrogallol per 3 ml assay mixture.

### Estimation of catalase (CAT) (E.C. 1.11.1.6)

CAT activity was measured by the method of Aebi [17]. To 1.95 ml of potassium phosphate buffer (50 mM, pH 7.0), 50 µl of sample was added; along with 1 ml of hydrogen peroxide (H_2_0_2_). The solution was read at 240 nm for 3 minutes. One enzyme unit is defined as the amount of enzyme decomposing 1 µM H_2_0_2_ per minute at 25°C.

### Estimation of glutathione-S-transferase (GST) (E.C 2.5.1.1.18)

GST activity was assayed in 0.2 M phosphate buffer (pH 6.5) after adding 1 mM 1-chloro 2, 4 dinitrobenzene (CDNB) and 1 mM GSH in the reaction mixture and following the increase of absorbance at 340 nm due to formation of the CDNB–GSH conjugate. One unit of enzyme activity is defined as the amount of enzyme catalyzing the formation of 1 µM product per min under the specific assay conditions [18]. Enzyme activity was expressed as units per mg of protein (molar extinction coefficient  = 9.6×103 M/cm).

### Estimation of glutathione (GSH)

The protocol of Jollow et al. [19] was essentially followed. Equal volume of homogenate was mixed with sulphosalicylic acid and incubated at 4°C for 1 hour followed by centrifugation at 1200 g for 15 minutes. The supernatant (0.2 ml) was taken and mixed with 1.1 ml of potassium phosphate buffer (0.1 M, pH 7.4). The reaction was initiated by the addition of 0.2 ml DTNB (5, 5′- dithiobis-2-nitrobenzoic acid) which was read at 412 nm within 30 seconds.

### Statistical analysis

Data was expressed as group mean ± SEM of three independent replicates of the experiment and analysed by one way ANOVA for differences between groups. P values less than 0.05 were considered statistically significant.

## Results and Discussion

Adult and larval trematode parasites infect vastly different hosts and adaptability seems to be a prerequisite for the perpetuation of the germ line. Using the model trematode parasite *C. complanatum*, experimentally established in markedly different hosts we studied levels of free radical scavengers during the course of maturation of this parasite.

**Figure 1:**
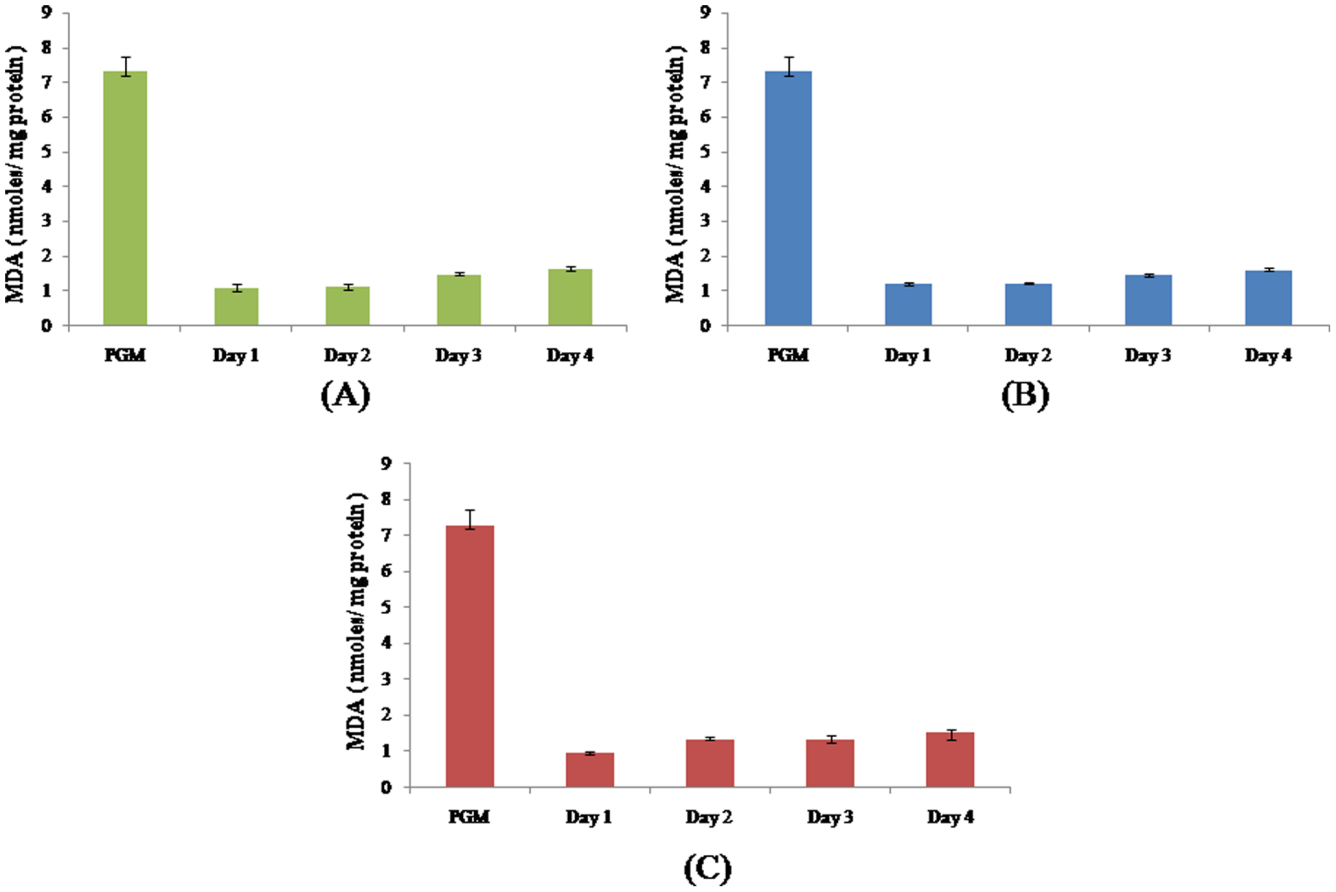
Lipid peroxidation during development of *Clinostomum complanatum*. Malionaldehyde levels (MDA) were estimated during the course of *Clinostomum complanatum* in chick buccal cavity (A), rabbit eye (B) and rat peritoneum (C). The data represent a mean of 3 independent experiments ± SEM. P≤0.05 when compared to Progenetic metacercaria (PGM) is considered significant in all cases.

As previously found in a study from our group [2], the progenetic metacercaria had significantly higher levels of lipid peroxidation as compared to the adult ovigerous worms (Fig. 1). There was a consistent increase in lipid peroxidation, consistently with the growth of the worm.

**Figure 2:**
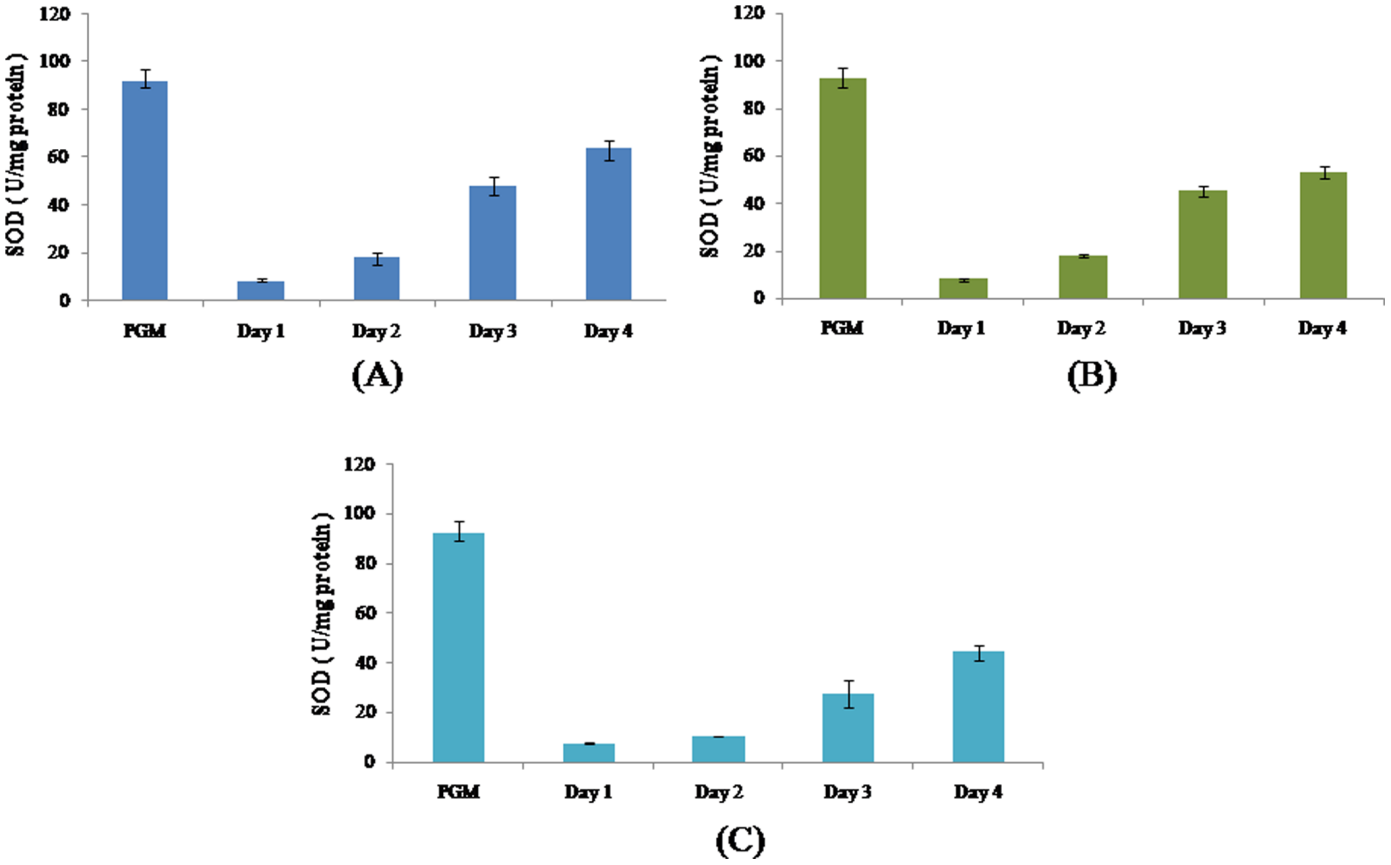
Superoxide dismutase levels during development of *Clinostomum complanatum*. SOD was estimated during the course of *Clinostomum complanatum* in chick buccal cavity (A), rabbit eye (B) and rat peritoneum (C). The data represent a mean of 3 independent experiments ± SEM. P≤0.05 when compared to Progenetic metacercaria (PGM) is considered significant in all cases.

**Figure 3:**
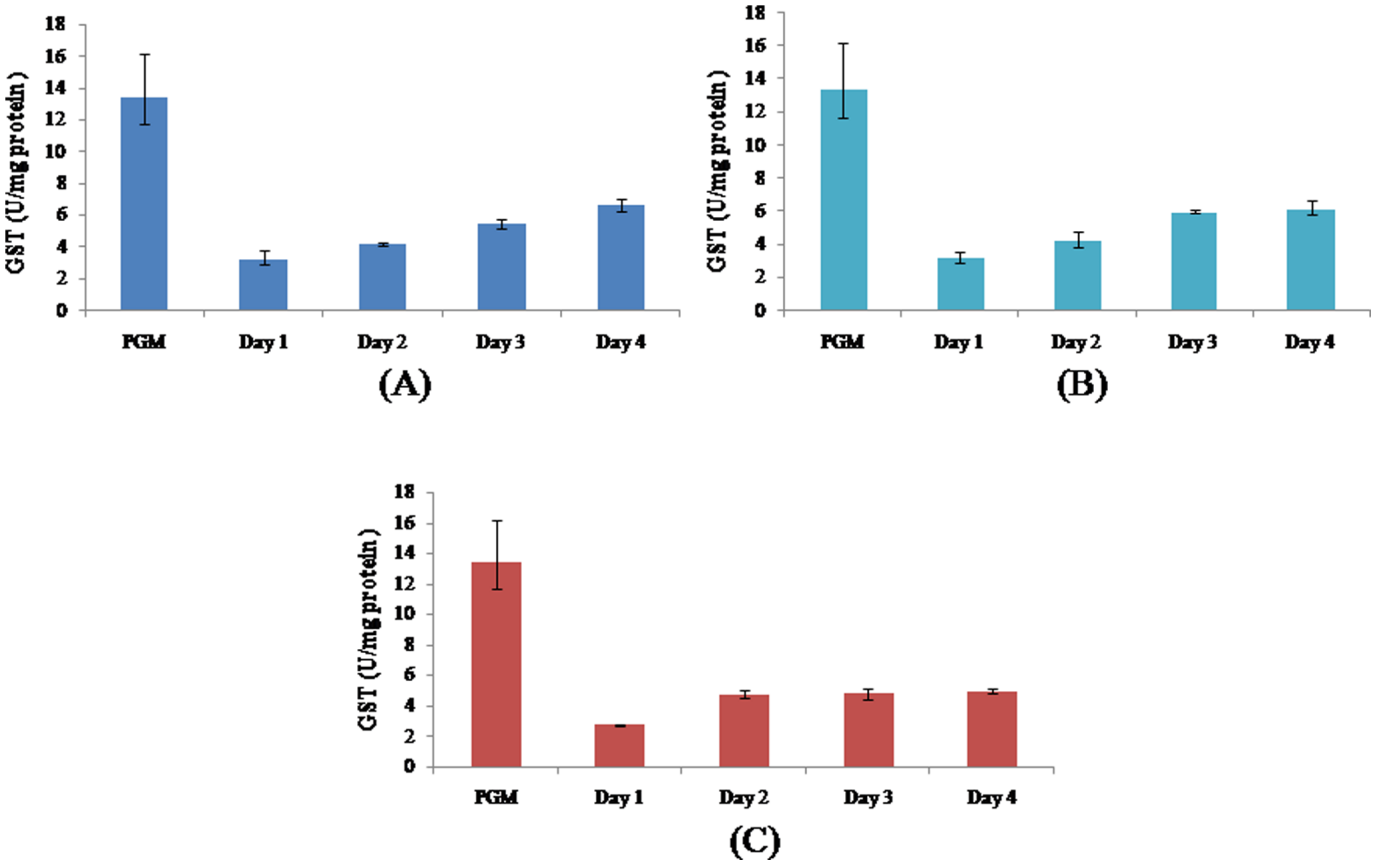
Glutathione-S-transferase (GST) levels during development of *Clinostomum complanatum*. (GST) was estimated during the course of *Clinostomum complanatum* in chick buccal cavity (A), rabbit eye (B) and rat peritoneum (C). The data represent a mean of 3 independent experiments ± SEM. P≤0.05 when compared to Progenetic metacercaria (PGM) is considered significant in all cases.

**Figure 4:**
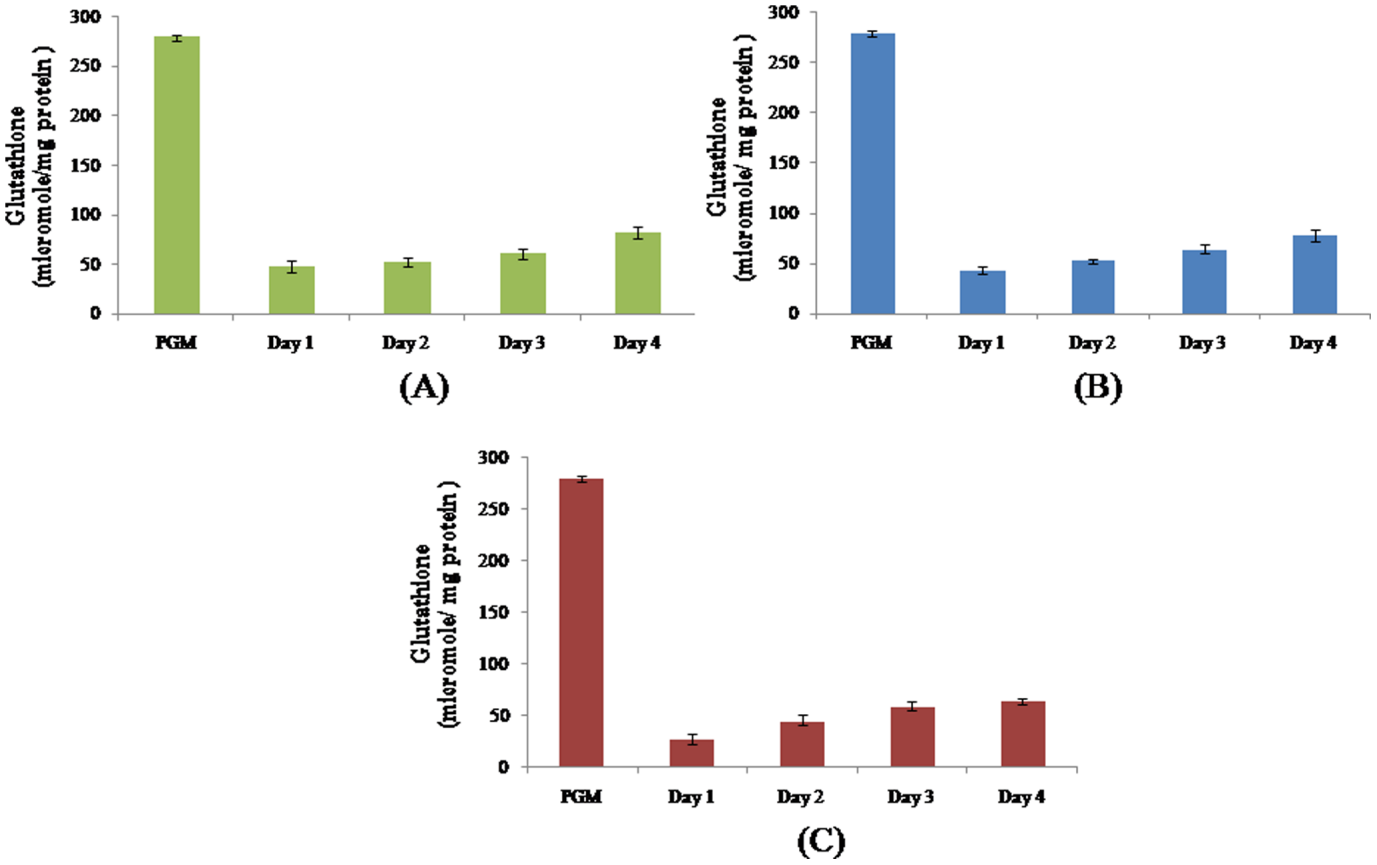
Glutathione levels during development of *Clinostomum complanatum*. Glutathione was estimated during the course of *Clinostomum complanatum* in chick buccal cavity (A), rabbit eye (B) and rat peritoneum (C). The data represent a mean of 3 independent experiments ± SEM. P≤0.05 when compared to Progenetic metacercaria (PGM) is considered significant in all cases.

All free radical scavengers evaluated in this study namely superoxide dismutase (Fig. 2), glutathione-S-transferase (Fig. 3) and glutathione (Fig. 4) were significantly elevated in the progenetic metacercaria as compared to the adult worms. In a previous communication by our group [2] we proposed that the progenetic metacercaria of *C. complanatum* stores free radical scavengers, and the developing worms utilize the available reserves of progenetic metacercaria, to combat free radicals produced by the host and remain within the definitive host (in the ovigerous state) only till the metacercarial supply of ROS scavengers lasts.

Our present results suggest that the free radical scavengers of the larva are only sufficient to combat the initial exposure to free radicals encountered by the parasite within the definitive host. We observe a consistent increase in the levels of superoxide dismutase, glutathione-S- transferase and glutathione as the worm progresses through its development (Fig. 2, 3, 4). It is plausible that for the worm to persist at the site of infection, the worm not only utilizes the available free radical scavenging reserves of the larval origin but also upon the influence of microenvironment at the site of infection (oxygen availability) produces its own free radical scavengers.

It is noteworthy that relative increase in the levels of superoxide dismutase, glutathione-S- transferase and glutathione observed during *in vivo* development corresponds to the availability of oxygen at the site of infection. This further strengthens the probability that the *de novo* synthesis of free radical scavengers occurs in response to both, the availability and the extent of availability of oxygen at the site of infection.

In all the developmental stages of the parasite and in all the three model hosts, no significant activity of catalase was detected. This is consistent with the previous reports [13], [20], [21]. It has been speculated that presence of secreted superoxide dismutase with the absence of catalase causes metabolic uncoupling and leads to conversion of superoxide free radical to hydrogen peroxide which is highly reactive and causes killing of immunity cells [22].

Since free radical scavengers of parasite origin play a crucial role in the developmental biology of trematodes, these molecules serve as putative vaccine targets to combat trematode infections.

The superoxide dismutase based vaccines SmCT-SOD, against Schistosomiasis has been shown to provide prophylactic protection against the disease [23]. Glutathione-S-transferase of the parasite origin is also being used as a vaccine target with considerable success [24].

This is the first ever study to report that the site of infection (and hence availability of oxygen) and the host, influence the levels of free radical scavengers of parasite origin. It is reasonable to presume that vaccines targeting free radical scavengers of parasite origin would show variable efficacy in response to ectopic infections.

Consistent with our hypothesis it has been shown that different host organisms respond differently to free radical scavenger based vaccines [25]. We propose that a major contributing factor for this effect is the differential levels of free radical scavengers produced by the same parasite in different hosts. Further studies are in progress in our laboratory to elucidate the mechanisms in response to which trematodes modulate their free radical scavengers.
